# Are North American Bunyamwera serogroup viruses etiologic agents of human congenital defects of the central nervous system?

**DOI:** 10.3201/eid0104.950409

**Published:** 1995

**Authors:** C H Calisher, J L Sever

**Affiliations:** Colorado State University, Fort Collins, Colorado, USA.


### Are North American Bunyamwera Serogroup Viruses Etiologic Agents of Human Congenital Defects of the Central Nervous System?

In 1941 Gregg provided the first evidence that rubella virus (family *Togaviridae*, genus *Rubivirus*) causes human congenital defects (1). Although rubella virus infection usually causes a mild disease comprising fever and rash, rubella epidemics have been associated with congenital defects in children of women who became infected during their first trimester of pregnancy (2). The risk of in utero rubella infection was reduced by the introduction of safe and effective vaccines for women of child-bearing age. Congenital abnormalities in fetal or neonatal ruminants also are related to exposure of pregnant dams to various viruses, including bovine viral diarrhea virus (family *Togaviridae*, genus *Pestivirus*), the arthropod-borne bluetongue viruses (family *Reoviridae*, genus *Orbivirus*), Wesselsbron virus (family *Flaviviridae*, genus *Flavivirus*), Rift Valley fever virus (family *Bunyaviridae*, genus *Phlebovirus*), Nairobi sheep disease virus (family *Bunyaviridae*, genus *Nairovirus*), and Akabane and Aino (family *Bunyaviridae*, genus *Bunyavirus*, Simbu serogroup) viruses (3-10). Infections of livestock with these viruses may produce low-titer viremia with no apparent clinical disease, or high-titer viremia and severe clinical illness in the dam. In utero infections may result in malformations of the developing fetus, fetal death with resorption, mummification, or miscarriage. Stillborn ruminants may show various musculoskeletal and central nervous system defects, including a syndrome of arthrogryposis with hydranencephaly (AGH).

Bunyamwera serogroup viruses (family *Bunyaviridae*, genus *Bunyavirus*) have been isolated from humans, and some, including Cache Valley (CV) and Tensaw (TEN) viruses, have been isolated from symptomatic and asymptomatic large mammals (11). Antibodies to CV virus and other viruses of the Bunyamwera serogroup are prevalent in livestock and large wild mammals and in humans in the Western Hemisphere from Alaska to Argentina (11). Viruses of this serogroup are isolated primarily from mosquitoes of the genera *Aedes* and *Anopheles*. These viruses have focal geographic distributions, although some are found over great expanses. CV virus, a common North American bunyavirus, has been isolated principally from mosquitoes of the genera *Culiseta*, *Aedes*, and *Anopheles*. The geographic distribution of this virus includes all North America, except the extreme southeastern states and southern Mexico (11). In the southeastern United States, TEN virus, also isolated from mosquitoes of the genera *Anopheles* and *Aedes*, is the only known representative of the Bunyamwera serogroup, probably because of the range of the principal vectors and vertebrate hosts; mutual exclusion of these two viruses likely occurs because of cross-protectivity between them (12).

Serologic and temporal associations of infection with CV virus and congenital malformations, including primarily AGH, were observed in sheep near San Angelo, Texas, between December 1986 and February 1987, suggesting that this virus causes AGH (13). Subsequent outbreaks of similar congenital defects occurred in sheep in Illinois in 1988 (J. Pearson, pers. comm.) and in North Dakota, Pennsylvania, Maryland, Michigan, and Nebraska in 1986 and 1987 (14). Antibody to CV virus (but not to other viruses) was found to correlate significantly with the occurrence of AGH and other congenital anomalies during the Texas outbreak (15, 16), and IgM antibody to CV virus was detected in colostrum-free neonates with AGH (C.H. Calisher, unpublished data). (Neither maternal IgM nor maternal IgG crosses the placenta in sheep; therefore, antibody in fetuses or in neonates before they received colostrum indicates fetal exposure to an infectious agent [17].)

In 1976, antibody to CV virus was detected in serum from cattle that had delivered calves with AGH in Saskatchewan, Canada, in 1975; however, the prevalence of antibodies to CV virus in the bovine population of that area was not investigated (R.E. Shope, pers. comm.). In Texas, in 1981, CV virus was isolated from a sick sheep and from a healthy cow in a herd with reproductive problems (18). This virus also was isolated in Texas in 1988 from a sentinel sheep in pasture where an outbreak of congenital defects had occurred in 1986 to 1987. These historical data suggest that

CV-virus-related congenital malformations may be more widespread than has been recognized.

Experimental infections have provided further evidence that CV virus causes embryonic death and multiple congenital malformations of sheep (19) and cattle (J. Edwards, pers. comm.). No association of TEN virus and disease in livestock, wild animals, or humans has been reported. To determine whether infection with selected Bunyamwera serogroup viruses, CV and TEN, is associated with certain human congenital defects, a serosurvey was done with serum samples from mothers of children with microcephaly or macrocephaly, and the results were compared with those from age- and location-matched controls. The results, reported here, provide the first evidence that these Bunyamwera serogroup viruses may be etiologic agents of certain congenital defects of the human central nervous system.

Two groups of 500 each human serum samples were selected at random from an archival collection stored at the National Institutes of Health (NIH), Bethesda, Maryland, and tested for neutralizing antibody. These samples were part of a collection of serum specimens obtained between 1959 and 1964, at delivery or post partum, (from about 50,000 women enrolled under defined protocols in a prospective study of congenital rubella syndrome. Specimens had been collected from all pregnant women cared for at the particular institution or from women randomly selected by using the last digit of their hospital registration number.

For a first series of tests, samples were selected from 200 mothers of children with macrocephaly (head size at least two standard deviations above the mean) and from 50 mothers of children with microcephaly (head size at least two standard deviations below the mean). The initial specimen from mothers with the respective defects was selected for testing. An equal number of mothers of babies without obvious central nervous system defects were selected as controls, which were age- (±2 years) and site-matched, were registered for the study in the same month, and were of the same race as mothers of children with either macrocephaly or microcephaly. Serum samples comprising this first group had been collected from pregnant women in Boston, Massachusetts (104), Providence, Rhode Island (20), New York, New York (72), Philadelphia, Pennsylvania (36), Baltimore, Maryland (38), Buffalo, New York (50), Minneapolis, Minnesota (42), Charlottesville, Virginia (38), Memphis, Tennessee (52), New Orleans, Louisiana (36), and Portland, Oregon (12); these were tested for neutralizing antibody to eight bunyaviruses (Table 1).

An additional 500 samples were tested; 250 paired samples, selected as above, from the same archival collection, two each from women in Boston (120), Buffalo (40), New Orleans (8), New York City (46), Baltimore (8), Charlottesville (12), and Minneapolis (16), including controls (selected as above). These were tested for neutralizing antibody to CV virus only, to follow up on results of tests with the first set of samples. One sample from each of these women had been collected in the first trimester of pregnancy, and the second had been collected at least 3 months later. Serum specimens were stored frozen at $-20^{o}C$ until they were shipped on dry ice ($-70^{o}C$) to the Centers for Disease Control laboratory at Fort Collins, Colorado.

Twenty-nine of the first 500 serum specimens tested contained neutralizing antibody to CV, 29 had antibody to TEN, 29 to Jamestown Canyon, 26 to La Crosse, nine to Lokern, and six to Buttonwillow viruses. None had antibody to Main Drain or Mermet virus. No significant differences were observed in antibody prevalences to La Crosse, Jamestown Canyon, Lokern, and Buttonwillow viruses between mothers of microcephalic and macrocephalic infants and age- and location-matched controls (Table 1). Cases were not reviewed for other defects.

The prevalence of antibody to CV virus in mothers of microcephalic infants was not significantly different from the prevalence of such antibody in their matched control, but the presence of antibody to CV virus in mothers was significantly correlated with macrocephaly in their infants ($\chi^2$, d.f. = 1, n = 400, 4.797 $p < 0.05$) (Table 1).

None of the samples with neutralizing antibody to CV or TEN virus (titers ranged from 10 to 80) had IgM antibody. To determine whether there were statistically significant differences between prevalences of antibody to CV or TEN virus in mothers of macrocephalic infants and in their age- and site-matched controls, McNemar's chi-square was used. No significant difference was found for the presence of antibody to CV virus ($p > 0.05$), but the presence of antibody to TEN virus ($p < 0.05$ or antibody to either CV or TEN virus ($p < 0.02$) was related to the occurrence of macrocephaly in the infants of these mothers (Table 2).

Table 1. Antibody to Cache Valley, Tensaw, La Crosse, Jamestown Canyon, Lokern, or Buttonwillow viruses in mothers of microcephalic or macrocephalic infants and matched controls

| Antibody to virus | Infant's condition | $\chi^2 (p)$ |
|---|---|---|
| Cache Valley | Microcephaly | 3.840 (0.05) |
| | Macrocephaly | 4.797 (<0.05) |
| | Either | 0.915 (>0.20) |
| Tensaw | Microcephaly | 4.891 (<0.05) |
| | Macrocephaly | 4.071 (<0.05) |
| | Either | 0.329 (>0.20) |
| Cache Valley or Tensaw | Microcephaly | 5.983 (<0.02) |
| | Macrocephaly | 4.806 (<0.05) |
| La Crosse | Microcephaly | 0.211 (>0.20) |
| | Macrocephaly | 1.481 (>0.20) |
| Jamestown Canyon | Microcephaly | 0.709 (>0.20) |
| | Macrocephaly | 0.037 (>0.20) |
| Lokern | Microcephaly | 1.010 (>0.20) |
| | Macrocephaly | 2.041 (>0.10) |
| Buttonwillow | Microcephaly | 2.041 (>0.10) |
| | Macrocephaly | <0.001 (>0.20) |

Bunyaviruses used for all tests were prototypes: (Bunyamwera serogroup) CV (strain 6V-633), TEN (A9-171b), Lokern (FMS-4332), Main Drain (BFS-5015), (California serogroup) La Crosse (Original), Jamestown Canyon (61V-2235), (Simbu serogroup) Buttonwillow (A-7956), and Mermet (AV-782). Samples were tested for antibody by serum dilution-plaque reduction neutralization (20). Briefly, diluted and heat-inactivated (56°C/30 min) serum was added to an equal volume of virus containing approximately 200 plaque-forming units (PFU), such that the final virus dilution was 100 PFU. Fresh human serum at a final concentration of 8% was added to all virus suspensions. Serum-virus mixtures were incubated at 4°C for 18 h, and 0.1-ml aliquots were dropped in the center of Vero cultures grown in 6-well plastic plates. After a 45-min period of adsorption, cells were overlaid with medium containing 2% agar and further incubated for 70 to 75 h at 37°C in 5% $CO_2$-95% air. A second overlay, containing medium, 2% agar, and 1:25,000 neutral red was then added, and the plates again were incubated until plaques were readable, usually 12 to 36 h later. A serum specimen was considered positive when it reduced the number of plaques 90% relative to control titrations, which ranged from 80 to 150 plaques. Samples that were positive in a screening (1:10) test were titrated for end-point, and the serum titer was taken as the highest twofold dilution of serum that reduced the number of plaques 90%.

Although a human positive control for detecting IgM antibody by capture enzyme-linked immunosorbent assays was not available, serum specimens were tested by a modification of a published technique for detecting IgM antibody to California serogroup viruses (21). Mouse or sheep serum samples containing IgM antibody to CV virus or mouse serum with IgM antibody to TEN virus served as positive and negative IgM antibody controls. Statistical analyses were done by chi-square or McNemar's chi-square.

Table 3 summarizes the presence of antibody to CV and to TEN virus in women by hospital location and birth outcome. When prevalence of antibody to CV or TEN virus in these women was analyzed by location and birth outcome, no statistically significant differences were determined (comparative data not shown).

When the second set of 500 specimens (250 paired early- and late-pregnancy samples) was tested, specimens from eight women had neutralizing antibody to CV virus. No diagnostically meaningful change in titer was detected between six sample pairs, but fourfold rises in titer were detected in two others, (10 to 80, <10 to 40), indicating recent infections with CV virus or with a closely related Bunyamwera virus group. Six of the eight women in this group with antibody to CV virus delivered macrocephalic infants, including the two (one in New Orleans and one in New York City) whose specimens showed rises in titer to CV virus.

These analyses provide the first evidence that Bunyamwera serogroup viruses in North America are associated with congenital defects in humans: the occurrence of macrocephaly in infants was positively correlated with antibody to CV virus. Antibody to TEN virus and to either CV or TEN virus correlated with microcephaly and with macrocephaly. The presence of antibody to CV and to TEN viruses corresponded with the known geographic distributions of these viruses within the United States. Antibody to either of these viruses in a woman living in an area where that virus is not known to occur may reflect the close antigenic relationships and considerable cross-reactivity of these Bunyamwera serogroup viruses (22), differences between local virus strains and prototype viruses, or travel to an area in which the virus does occur (23). The two women with diagnostically significant rises in antibody titer to CV virus (one from New Orleans, where TEN virus has been isolated, and one from New York, where CV virus has been isolated) are, as far as we know, the first two persons with documented rises in antibody to a Bunyamwera serogroup virus in North America. Whether either of them had an associated illness could not be determined from the records. That among all the women tested they were the only ones with rises in antibody titer and that both gave birth to macrocephalic infants is, at the least, a fascinating coincidence. IgM antibody in human infections caused by other bunyaviruses may not persist for much more than a few months after

Table 2. Presence of antibody to Cache Valley and Tensaw viruses in mothers of macrocephalic infants and in matched controls

| Mothers of macrocephalic infants | No. | Antibody-positive controls | Antibody-negative controls |
|---|---|---|---|
| Antibody to CV virus | 16 | 3 | 13 |
| No antibody to CV virus | 184 | 5 | 179 |
| McNemar's test[a] | | | |
| Antibody to TEN virus | 15 | 1 | 14 |
| No antibody to TEN virus | 185 | 5 | 180 |
| McNemar's test[b] | | | |
| Antibody to CV or TEN virus (or both) | 19 | 3 | 16 |
| No antibody to CV or TEN virus | 181 | 5 | 176 |
| McNemar's test[c] | | | |

[a] $(13-5)^2/18 = 3.556$ (d.f. = 1), $p > 0.05$. [b] $(14-5)^2/19 = 4.263$ (d.f. = 1), $p < 0.05$. [c] $(16-5)^2/21 = 5.762$ (d.f. = 1), $p < 0.02$.

infection (21); IgM antibody has not been detected in humans with antibody to Bunyamwera serogroup viruses in North America. Therefore, the absence of IgM antibody in specimens with neutralizing antibody likely indicates that these infections were not acute, i.e., they occurred months before the specimens were collected.

A fundamental problem in establishing a relationship between infection of mammals with CV or TEN virus and attendant congenital anomalies is the inherent inadequacy of controls. The presence of antibody in humans or other animals with normal offspring does not necessarily argue against the hypothesis that CV virus causes congenital defects in humans because infection could have occurred before pregnancy. Additionally, even if this virus can be an etiologic agent of congenital anomalies, preexisting antibody to this virus could provide immunity for the mother and protect the fetus from viral infection. Thus, it cannot be determined with certainty whether the presence of antibody to a virus is coincidental to, or a cause of, the observed congenital anomalies.

Review of NIH records for this relatively small set of samples suggested that macrocephaly occurred somewhat more often when the first trimester of pregnancy included the months April and May for women living in New Orleans (4/16), Memphis (5/16), and Charlottesville (3/12) and in late summer-early autumn for women living in Boston (6/49), Minneapolis (3/11), Portland (2/6), and New York City (9/33). In each instance, these periods coincide roughly with the appearance of populations of *Culiseta*, *Aedes*, or *Anopheles* mosquitoes, the vectors of CV and TEN viruses. However, CV virus cannot be implicated in infections in New Orleans because this virus is not known to occur there, although TEN virus does.

The relatively small sample sizes in this study allow statistical interpretation but do not provide sufficient support to warrant statements as to the biological significance of the findings; therefore, we consider these data merely preliminary. Determining whether these data have merit awaits the results of additional studies of mothers of children with congenital defects and their offspring. More extensive studies are also needed to investigate the influence of gestational phase and fetal development on congenital defects; establish relationships between peak abundance of arthropod vectors and first trimesters of pregnancies; sequence the genomes of CV and TEN virus strains from various geographic areas and establish relationships between different gene sequences and virulence in livestock; and develop diagnostic capacity by using monoclonal antibodies, hybridization assays, polymerase chain reaction, and Western blotting techniques.

Table 3. Antibody to Cache Valley and Tensaw viruses in women, by location and birth outcome

| | Antibody to CV virus | | Antibody to TEN virus | |
|---|---|---|---|---|
| Location | In women with macrocephalic infants:: no. (%) | In controls: no. (%) | In women with macrocephalic: infants: no. (%) | In controls: no. (%) |
| Boston | 2/49 (4.1) | 1/49 (2.0) | 1/49 (2.0) | 1/49 (2.0) |
| Providence | 0/7 | 1/7 (14.3) | 0/7 | 1/7 (14.3) |
| New York City | 2/33 (6.1) | 1/33 (3.0) | 1/33 (3.0) | 1/33 (3.0) |
| Philadelphia | 0/15 | 2/15 (13.3) | 1/15 (6.7) | 2/15 (13.3) |
| Minneapolis | 1/11 (9.1) | 0/1 | 1/11 (9.1) | 0/11 |
| Charlottesville | 2/12 (16.7) | 0/12 | 2/12 (16.7 | 0/12 |
| Memphis | 6/16 (37.5) | 3/16 (18.8) | 5/16 (31.3) | 1/16 (6.3) |
| New Orleans | 1/16 (6.3) | 0/1 | 2/16 (12.5) | 0/16 |
| Portland | 2/6 (33.3) | 0/6 | 2/6 (33.3) | 0/6 |

Given that many members of the family *Bunyaviridae* cause congenital defects in naturally and experimentally infected livestock, or may have such a potential (24,25), it would be worthwhile to continue investigations with domestic animals and to develop laboratory models to assess the teratogenic potential for humans of CV, TEN, and other viruses of the family *Bunyaviridae*.

It is also important to determine the roles of CV and TEN viruses in inducing human congenital defects and the relationships between prevalence of antibody to CV and TEN viruses, prevalence of congenital defects, and conception dates, all with respect to local environmental conditions.

**Charles H. Calisher, Ph.D.,* and John L. Sever, M.D.†**
*Colorado State University, Fort Collins, Colorado, USA;
†George Washington University Medical Center, Children's National Medical Center, Washington, D.C., USA

### Acknowledgments

The authors thank Dr. Maneth Gravell and Ms. Dorothy O'Neill, National Institutes of Health, Bethesda, Maryland, for selecting, sorting, and shipping serum samples; Mr. Raymond E. Bailey, Centers for Disease Control and Prevention, Fort Collins, Colorado, for statistical advice; Dr. Thomas P.C. Monath, Division of Vector-Borne Infectious Diseases, CDC, Fort Collins, for his encouragement and support; and Drs. Barry Beaty, Colorado State University, Fort Collins, and John Edwards, Texas A&M University, College Station, Texas, for their editorial suggestions.

### References

1. Gregg N McA. Congenital cataract following German measles in the mother. Trans Ophthal Soc Austral 1941;3:35-46.
2. Best JM, O'Shea S. Rubella virus. In: Schmidt NJ, Emmons RW, editors. Diagnostic procedures for viral, rickettsial and chlamydial infections, 6th ed. Washington, DC: American Public Health Association 1989:731-95.
3. Oberst RD. Viruses as teratogens. Vet Clin North Am; Food Anim Prac 1993:9:23-31.
4. Erasmus BJ. The history of bluetongue. In: Barber TL, Jochim MM, editors. Bluetongue and related orbiviruses. New York: AR Liss, 1985:7-12.
5. Calisher CH, Monath TP. *Togaviridae* and *Flaviviridae*: the alphaviruses and flaviviruses. In: Lennette EH, Halonen P, Murphy FA, editors. Laboratory diagnosis of infectious diseases, vol. 2. New York: Springer-Verlag, 1988:414-34.
6. Calisher CH, Shope RE. Bunyaviridae: the bunyaviruses. In: Lennette EH, Halonen P, Murphy FA, editors. Laboratory diagnosis of infectious diseases, vol. 2. New York: Springer-Verlag, 626-46.
7. Kurogi H, Inaba Y, Takahashi E, Sato K, Omori T, Miura Y, et al. Epizootic congenital arthrogryposis-hydranencephaly syndrome in cattle: isolation of Akabane virus from affected fetuses. Arch Virol 1976;51:67-74.
8. Kurogi H, Inaba Y, Takahashi E, Sato K, Satoda K, Goto Y, et al. Congenital abnormalities in newborn calves after inoculation of pregnant cows with Akabane virus. Infect Immun 1977:17:338-43.
9. Parsonson IM, Della-Porta AJ, Snowdon WA, Murray MD. Congenital abnormalities in foetal lambs after inoculation of pregnant ewes with Akabane virus. Austral Vet J 1975:51:585-6.
10. Coverdale OR, Cybinski DH, St. George TD. Congenital abnormalities in calves associated with Akabane and Aino virus. Austral Vet J 1978:54:151-2.
11. Calisher CH, Francy DB, Smith GC, Muth DJ, Lazuick JS, Karabatsos N, et al. Distribution of Bunyamwera serogroup viruses in North America, 1956-1984. Am J Trop Med Hyg 1986;35:429-43.
12. Calisher CH. Evolutionary significance of the taxonomic data regarding bunyaviruses of the family *Bunyaviridae*. Intervirology 1988;29:268-76.
13. Crandell RA, Livingston CW. Laboratory investigation of a naturally occurring outbreak of arthrogryposis-hydranencephaly in Texas sheep. J Vet Diagn Invest 1988;1:62-5.
14. Rook JS, Yamini B, Steficek B. AGH syndrome: cooperation answers questions. National Wool Grower 1988(April):24-5.
15. Edwards JF, Livingston CW, Chung SI, Collisson EC. Ovine arthrogryposis and central nervous system malformations associated with in utero Cache Valley virus infection: spontaneous disease. Vet Pathol 1989;26:33-9.
16. Edwards JF. Cache Valley virus. Vet Clin North Am; Food Anim Prac 1994;10:515-24.
17. Campbell SG, Siegel MJ, Knowlton BJ. Sheep immunoglobulins and transmission to the neonatal lamb. N Z Vet J 1977;25:361-5.
18. McConnell S, Livingston C Jr, Calisher CH, Crandell R. Isolations of Cache Valley virus in Texas, 1981. Vet Microbiol 1987;13:11-8.
19. Chung SI, Livingston CW Jr, Edwards JF, Gauer BB, Collisson EW. Congenital malformations in sheep resulting from in utero inoculation of Cache Valley virus. Am J Vet Res 1990;51:1645-8.
20. Lindsey HS, Calisher CH, Mathews JH. Serum dilution neutralization test for California group virus identification and serology. J Clin Microbiol 1976;4:503-10.
21. Calisher CH, Pretzman CI, Muth DJ, Parsons MA, Peterson ED. Serodiagnosis of La Crosse virus infections in humans by detection of immunoglobulin M class antibodies. J Clin Microbiol 1986;23:667-71.
22. Calisher CH, Lazuick JS, Lieb S, Monath TP, Castro KG. Human infections with Tensaw virus in south Florida: evidence that Tensaw virus subtypes stimulate the production of antibodies reactive with closely related Bunyamwera serogroup viruses. Am J Trop Med Hyg 1988;39:117-22.
23. Calisher CH, Sabattini MS, Wolff KL, Monath TP. Cross-neutralization tests of South American isolates of Cache Valley virus revealing the existence of multiple subtypes. J Trop Med Hyg 1988;39:202-5.
24. Parsonson IM, Della-Porta AJ, Snowdon WA. Developmental disorders of the fetus in some arthropod-borne virus infections. Am J Trop Med Hyg 1981;30:660-73.
25. McPhee DA, Parsonson IM, Della-Porta AJ, Jarrett RG. Teratogenicity of Australian Simbu serogroup and some other Bunyaviridae viruses: the embryonated chicken egg as a model. Infect Immun 1984;43:413-20.
